# Ocular blood flow in preterm neonates

**DOI:** 10.1038/s41598-024-58523-8

**Published:** 2024-04-02

**Authors:** Ronald H. Silverman, Raksha Urs, Jason D. Horowitz, Osode Coki, Leora Pinto

**Affiliations:** 1 Department of Ophthalmology, Columbia University Irving Medical Center, New York, NY USA; 2 Department of Ophthalmology, Columbia University Irving Medical Center, 701 West 168th St., Room 609B, New York, NY 10032 USA

**Keywords:** Retinopathy of prematurity, Medical research, Translational research

## Abstract

Retinopathy of prematurity (ROP) is a disorder affecting low birthweight, preterm neonates. In the preterm eye, the retina is not fully developed and neovascularization may occur at the margin between the developed vascular retina and undeveloped avascular retina. Without timely treatment by laser or intravitreal anti-vascular endothelial growth factor (VEGF) therapy, this can lead to tractional retinal detachment and blindness. Visualization of the retina in regular examinations by indirect ophthalmoscopy is hence the current standard of care, but the exams are stressful and interpretation of images is subjective. The upregulation of VEGF in ROP would suggest an increase in ocular blood flow. In this report, we evaluate the potential of ultrafast plane-wave Doppler ultrasound (PWU) to detect increased flow velocities in the orbital vessels supplying the eye in a gentle exam with objective findings. We imaged both eyes of 50 low-birthweight preterm neonates using 18 MHz PWU. Flow velocity in the central retinal artery (CRA) and vein (CRV), and the short posterior ciliary arteries were determined and values at each ROP Stage compared. We found significantly increased velocities in the CRA and CRV in Stage 3 ROP eyes, where intervention would be considered. We compared multivariate models for identifying Stage 3 eyes comprised solely of clinical factors, solely of Doppler parameters, and clinical plus Doppler parameters. The respective models provided areas under their respective ROC curves of 0.760, 0.812, and 0.904. PWU Doppler represents a gentle, objective means for identifying neonates at risk for ROP that could complement ophthalmoscopy.

## Introduction

Retinopathy of prematurity (ROP) is a potentially devastating disorder of retinal vascular development occurring in low birthweight, preterm neonates. In spite of advances in neonatal intensive care, ROP remains a leading cause of pediatric blindness and potential lifelong visual impairment^1^. ROP incidence among premature infants in the United States has increased, especially among the Black and Hispanic populations^2^.

Starting at about 16 weeks gestation, the retina develops from the optic nerve outwards, a process that is complete at about 40 weeks^3^. The retinal vasculature is thus incompletely developed and immature in preterm neonates.

Oxygen plays a central role in retinal vessel development^4,5^. In utero, ‘physiological hypoxia’ allows normal retinal angiogenesis, primarily at the retinal periphery^6,7^, with VEGF playing a key role^8^. After preterm delivery and exposure to higher oxygen levels, there is cessation of vessel development and loss of already formed vessels. The avascular retina becomes more metabolically active and VEGF is upregulated; pathologic neovascularization with the potential for retinal detachment then follows^7^.

The current standard of care for preterm neonates entails examination of the eye by indirect ophthalmoscopy or wide-angle contact photography (RetCam, Natus Medical, Pleasanton, CA) on a regular basis. The first eye screening exam is appropriate at 4 weeks of age for neonates with gestational age (GA) of 27 weeks or more and at up to 9 weeks for progressively lower GA. Follow-up exams may occur weekly or less frequently, depending on ROP Stage and Zone observed in the prior exam^9^. ROP stages^10,11^ and their characteristics are outlined in Table 1.

**Table 1:** ROP Stages and their characteristics.

ROP Stage	Characteristics
0	No clear demarcation line between vascularized and non-vascularized retina
1	Demarcation line is visible
2	Demarcation line has developed into a ridge
3	Blood vessels are visible at the ridge
4	Sub-total retinal detachment
5	Total retinal detachment

Expertise in performing indirect ophthalmoscopy, wide-angle digital imaging and image interpretation play a crucial role in ROP management, yet image interpretation is subjective. Examiners employing the latest RetCam imaging disagree 40% of the time as to even the presence or absence of ROP from its earliest stages to preplus and plus disease (i.e., abnormal retinal blood vessel tortuosity and dilation)^12–14^. There is thus a necessity for imaging methods offering objective, reproducible diagnostic criteria^11,12,15,16^.

Ophthalmoscopic examination of preterm neonates at risk for ROP has significant limitations beyond subjectivity: Exposure to mydriatic agents, bright light and use of a lid speculum and Flynn scleral depressor to conduct a detailed ophthalmoscopic or RetCam examination of the retina is stressful for the neonate, and not without potential morbidity^17–21^.

Optical coherence tomography (OCT) and OCT-angiography (OCTA) have been explored for examination of the preterm eye. They are advantageous with respect to ophthalmoscopy in not requiring exposure to bright lights or scleral depressors and the exam can be performed via the non-dilated pupil (although dilation is advantageous)^22–24^. OCT, however, is limited to the posterior pole of the eye and provides only structural information. OCTA offers *en face* mapping of the retinal vasculature, and thus assessment of vessel tortuosity, density, and non-perfused areas (e.g., foveal avascular zone area). OCTA, however, is impeded due to eye motion during imaging (> 3 s)^25,26^.

Unlike ophthalmoscopy, ultrasound does not require mydriatics and unlike OCT can readily image the posterior structures of the eye even through a closed eyelid. Conventional B-scan, however, has relatively poor resolution compared to optical methods and offers no information on retinal vascular structure or blood flow. Hence, the use of B-scan ultrasound in ROP has been limited, with most reports citing its use for detection of retinal detachment^27–30^. Jokl et al*.* evaluated the potential of 10–20 MHz ultrasound B-scan to provide diagnostically significant information, reporting detection of the ridge between vascularized and avascular retina^31^ and development of fibrovascular vitreous membranes portending retinal detachment^32^.

All of the above techniques image structure rather than function. The central role of VEGF upregulation in ROP, however, suggests that ocular blood flow might be increased in ROP. Doppler ultrasound has thus been used to study ocular blood flow, a functional property, in a number of ROP studies, with variable results. Some reports found no differences between ROP and non-ROP eyes^33^ or in Stage 1–2 versus non-ROP^34^. Others reported significantly increased flow velocities in Stage-2 ROP^35^.

In the conventional Doppler systems utilized in the above studies, a focused ultrasound beam produced by a linear array is scanned to produce two-dimensional B-scan images. At each image position, the instrument must wait for echo data to be received before moving to the next position, which limits imaging speed to hundreds of B-scans per second. In pulsed Doppler, frequency shifts at specific ranges along the lines of sight are measured in real time to determine flow velocity, but with acoustic intensity exceeding FDA guidelines for ophthalmic imaging^36^.

**Figure 1:**
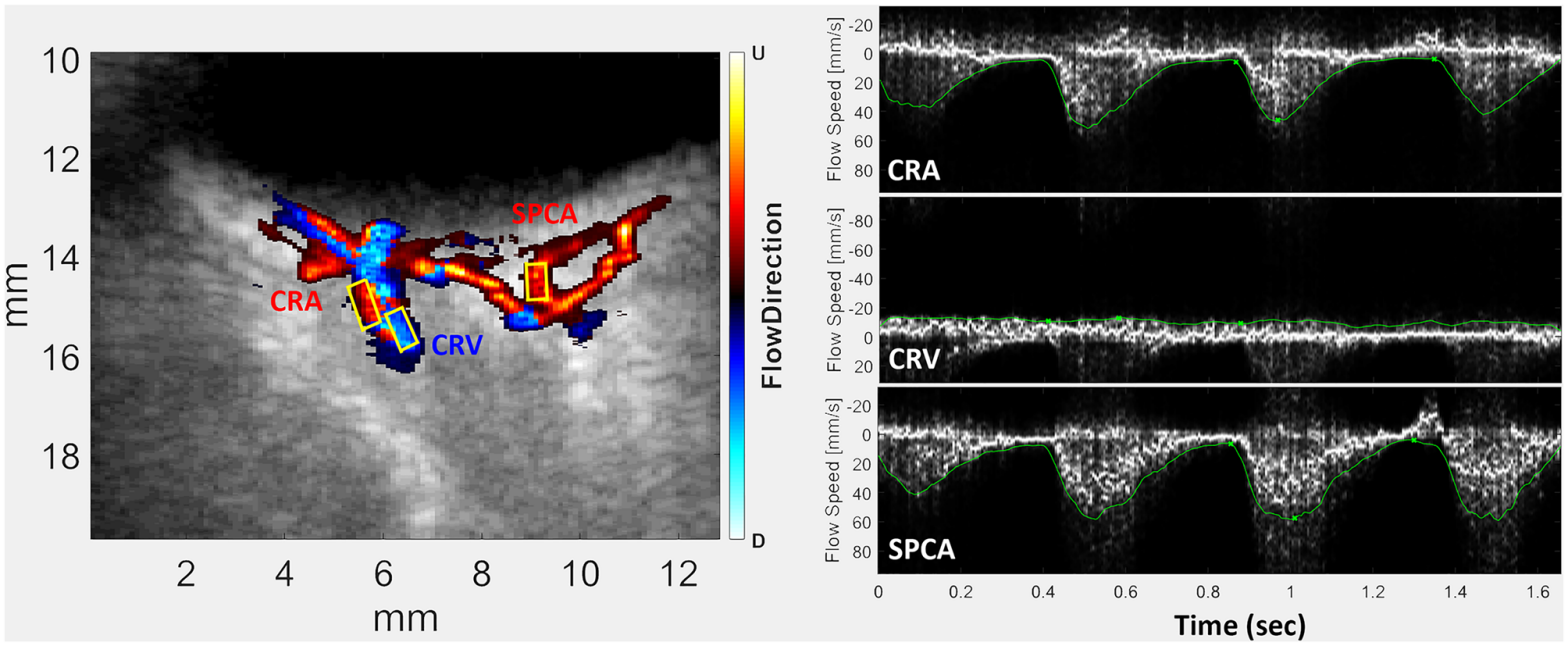
Left: Representative plane-wave color flow Doppler image of neonatal posterior pole. Right: Spectrograms of the CRA, CRV and SPCA.

Plane wave ultrasound (PWU) is an alternative approach that we utilized in a pilot study of 14 preterm neonates where we found increased arterial and venous flow velocities in Stage 3 ROP^37^. In PWU^38^, all the piezoelectric elements of a linear array act together to transmit an unfocussed wavefront. A focused image is created using a ‘delay-and-sum’ algorithm that brings echoes reaching each array element into phase by triangulating the time-of-flight from each image position. A B-scan image is thus formed with each transmit, allowing an imaging rate of over 20,000 B-scans/sec. Combining echo data from a series of angled transmits improves sensitivity and resolution^39^. By recording scans continuously over a cardiac cycle, Doppler analysis can be performed at any pixel position and systolic and diastolic flow velocities determined in post-processing. Furthermore, because the transmitted wavefront is unfocused, acoustic intensity is significantly less than would occur using a conventionally scanned, focused beam. PWU Doppler can be performed in compliance with ophthalmic regulatory standards, which is not the case in studies performed with conventional pulsed Doppler systems. This is a particularly important consideration in imaging the neonatal eye^40^. A representative PWU Doppler image and spectrograms depicting flow over the cardiac cycle is provided in Fig. 1.

## Results

Table 2 provides a breakdown of the study population characteristics. ROP Stage at the time of exams is provided in Table 3.

**Table 2:** Characteristics of neonates.

Factor	Mean ± SD	Range
Sex	M:76; F:86	
Birthweight (g)	761 ± 176	410–1370
Systolic blood pressure (mmHg)	78.9 ± 12.3	54–108
Diastolic blood pressure (mmHg)	48.0 ± 12.6	24–76
Mean arterial pressure (mmHg)	58.3 ± 11.4	38.3–85.7
Heart rate (BPM)	143.4 ± 33.5	57–193
Gestational age (weeks)	26.0 ± 2.0	20.2–30.2
Post-menstrual age at exam 1 (weeks)	37.2 ± 3.2	31.5–47.3
Post-menstrual age at exam 2 (weeks)	39.3 ± 4.1	32.3–50.2

**Table 3:** ROP Stage at initial and follow-up exams.

Stage	Initial exam		Follow up	
	N eyes	%	N eyes	%
0	34	34.0	22	35.5
1	15	15.0	0	0
2	41	41.0	31	50.0
3	10	10.0	9	14.5
Total	100	100	62	100

Of the clinical factors, only birthweight (BW) and GA were found to differ significantly by Stage (p < 0.001 by ANOVA). Both factors were significantly different with respect to Stage 0 eyes at Stages 2 and 3 (p < 0.05) by post-hoc Dunnet’s test.

The within exam standard deviations in the CRA were 12.2, 3.5 and 6.7 mmHg for the PSA, EDV and mean velocity respectively, corresponding to coefficients of variation of 0.28, 0.35 and 0.29. Variability in the CRV and SPCA were comparable.

ANOVA (Table 4) showed significant differences in Doppler flow velocity by Stage in the CRA and CRV. Flow velocities were significantly elevated in Stage 3.

**Table 4:** ANOVA of flow velocity by Stage for each vessel.

Vessel	Stage	N eyes	PSV (mm/s)	EDV (mm/s)	MV (mm/s)
CRA	0	55	38.2 ± 11.7	9.4 ± 3.6	20.6 ± 6.5
	1	13	42.9 ± 12.6	9.4 ± 3.3	22.2 ± 7.5
	2	70	40.6 ± 13.5	9.0 ± 2.9	21.6 ± 7.3
	3	19	60.5 ± 20.3**	14.0 ± 10.8**	33.6 ± 13.2**
	F		12.9	5.7	13.6
	p		< 0.001	0.001	< 0.001
CRV	0	54	17.7 ± 5.6	12.8 ± 4.8	14.7 ± 5.0
	1	13	17.3 ± 4.5	13.1 ± 4.3	14.4 ± 4.8
	2	71	16.3 ± 5.1	12.2 ± 4.3	13.9 ± 4.5
	3	19	28.3 ± 12.2**	20.5 ± 9.2**	24.1 ± 10.6**
	F		17.6	13.0	16.6
	p		< 0.001	< 0.001	< 0.001
SPCA	0	56	46.3 ± 14.0	13.1 ± 6.2	26.4 ± 9.6
	1	15	50.5 ± 19.6	12.7 ± 4.9	27.3 ± 11.3
	2	72	49.7 ± 13.7	13.4 ± 5.6	28.2 ± 9.3
	3	19	56.7 ± 14.4*	15.9 ± 7.8	32.6 ± 9.5*
	F		2.5	1.2	2.0
	p		0.062	0.328	0.119

*Indicates p ≤ .05 and **indicates p ≤ 0.01 by post-hoc Dunnett’s test with respect to Stage 0.

Eleven eyes had preplus disease: 2 at Stage-1, 4 at Stage-2 and 5 at Stage-3. There were only 4 Zone-I (region surrounding the optic nerve head) eyes in the study population. Flow velocities were not significantly altered by presence of preplus or Zone-I disease, although this is uncertain given the small numbers of either preplus or Zone-I.

Followup between exams ranged from 14 to 27 days, averaging 21 days. Between initial and followup exams, eight eyes progressed from Stage 1 to Stage 2 and one from Stage 2 to Stage 3. Flow velocity in the CRA and SPCA increased in this group (e.g., by 7.1 ± 15.7 mm/s PSV in the CRA), but given the small number of cases, this was not statistically significant.

Because flow velocities were comparable at Stages 0–2 and increased only at Stage 3, we compared Stage 3 with combined Stages 0–2. ANOVA results comparing flow in Stage 3 versus Stages 0–2 in both the initial exam and follow-up exam are presented in Table 5.

**Table 5:** One-way ANOVA for Stage 3 versus Stages 0–2 for all exams, initial exam only and follow-up exams only.

Vessel	Parameter	All exams		Initial exam		Follow-up exam	
		F	p	F	p	F	p
CRA	PSV	37.2	< 0.001	7.0	0.009	43.0	< 0.001
	EDV	17.0	< 0.001	0.2	0.652	20.6	0.018
	VM	40.4	< 0.001	6.2	0.014	46.1	< 0.001
CRV	PSV	51.6	< 0.001	17.5	< 0.001	40.0	< 0.001
	EDV	38.8	< 0.001	17.8	< 0.001	21.8	0.004
	VM	49.8	< 0.001	18.8	< 0.001	33.3	< 0.001
SPCA	PSV	5.4	0.021	0.5	0.515	10.7	0.002
	EDV	3.3	0.072	0.1	0.831	8.0	0.006
	VM	4.9	0.028	0.2	0.644	12.4	< 0.001

Follow-up exams were performed in approximately 60% of subjects, with dropouts attributable to patient discharge. A noteworthy observation is that in Stage 3 ROP mean flow velocity in the CRA increased on followup by 8.1 ± 20.1 mm/s whereas, the change was only − 0.3 ± 9.9 mm/s in Stages 0–2. Although not statistically significant, this increase might be attributable to progression in Stage 3 eyes.

**Figure 2:**
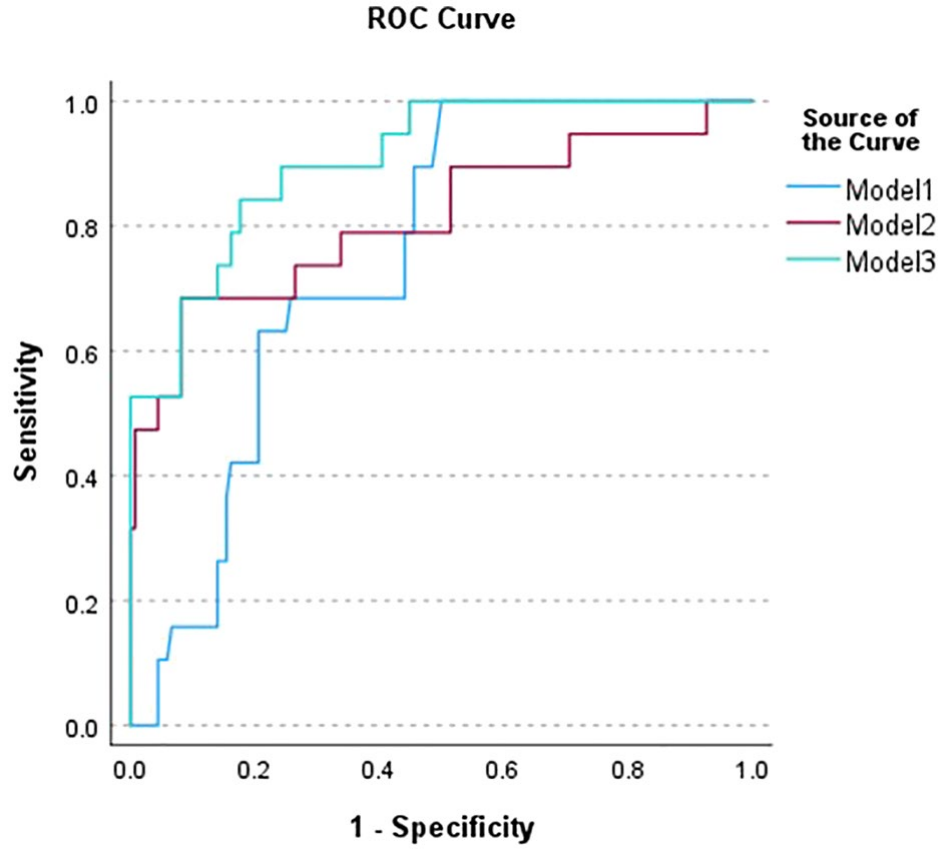
ROC curves for the clinical Model 1, Doppler Model 2, and Doppler plus clinical Model 3.

Clinical, Doppler and combined clinical + Doppler multivariate classification models are described in Table 6. Their ROC curves are plotted in Fig. 2 and the ROC area under curve for all models are shown in Table 6.

**Table 6:** Discriminant analysis models including only clinical parameters (Model 1), only Doppler parameters (Model 2) and Doppler plus clinical parameters (Model 3).

Parameter	Vessel	Standardized coefficient		
		Model 1	Model 2	Model 3
GA		− 0.822		− 0.417
Diastolic BP		0.624		0.327
MV	CRA		0.488	0.449
PSV	CRV		0.691	0.670
PI	SPCA		0.316	0.311
Correctly classified		64.8%	83.2%	83.9%
Correctly classified validation		64.8%	83.2%	83.9%
AUC		0.760 ± 0.044	0.825 ± 0.060	0.904 ± 0.034
Significance (two-tailed) with respect to Model 1		–	0.417	0.015

Standardized coefficients represent the weight of each parameter in models.

## Discussion

The variability of individual measurements was significant, with coefficients of variability of around 30%. This was ameliorated by taking three scans per eye, reducing effective uncertainty of measurement of individual eyes by a factor of √3, or 1.73.

We found flow velocities in Stage 1–2 ROP to not differ significantly from Stage 0 eyes, which is consistent with prior studies using pulsed Doppler ultrasound^34,35^. We also confirmed our preliminary finding of significantly increased flow velocity at Stage 3^37^.

When comparing blood pressure in Stage 3 versus Stages 0–2, no measures reached statistical significance as univariate parameters, although diastolic blood pressure was borderline elevated (p = 0.056, two-tailed) in Stage 3 (53.3 ± 11.6 mmHg versus 47.4 ± 12.6 mmHg in Stages 0–2), and entered into the multivariate stepwise models. We have no hypothesis as to an underlying mechanism, and this remains to be verified in a larger study.

The ROC curves of the multivariate models indicate the tradeoff between sensitivity and specificity that would need to be considered in staging of ophthalmoscopy. We would ideally like a classifier offering 100% sensitivity and specificity for Stage 3 detection, but the best model only achieves perfect sensitivity at 50% specificity, which may represent an unacceptable false-positive rate. At 90% sensitivity, however, the combined model provides ~ 75% specificity, which may represent a practical tradeoff.

Since treatment is not normally performed prior to Stage 3, these findings are potentially significant in terms of management: if Stage 3 could be detected by PWU, confirmation by ophthalmoscopy would be prioritized and conversely, if PWU does not indicate Stage 3 ROP, stressful ophthalmoscopic examinations could be performed less frequently.

There are several limitations to this study: Only 11 eyes were preplus and only 4 Zone-I; no patients were imaged pre- and post-treatment by laser or intravitreal anti-VEGF agents; Doppler measurements determined flow velocity, not volumetric flow; ophthalmoscopically determined ROP Stage and presence of preplus disease were treated as ground truth but are themselves subjective.

While the PWU Doppler technology utilized in this study utilized customized software and was implemented on a research ultrasound platform, turnkey PWU systems are now becoming available (albeit not configured for ophthalmology). It is probable that systems suitable for pediatric ophthalmology will in the future offer safe and gentle assessment of ocular blood flow so that these findings could impact ROP management.

In conclusion, PWU could be used to reduce the number of stressful ROP screening dilated ophthalmoscopic exams that babies would have to undergo. PWU could be combined with additional new screening algorithms to implement even further reduction. Possibly the blood flow measurements could contribute to prognostication and refinement of indications for treatment in combination with ophthalmoscopy and/or photography.

## Methods

This research followed the tenets of the Declaration of Helsinki and was approved by the Columbia University Institutional Review Board as well as by a separate neonatal intensive care unit (NICU) research ethics and safety committee. Informed consent was obtained from the parents after explanation of the nature, risks, and benefits of the study.

Neonate subjects were inpatients in the New York-Presbyterian/Morgan Stanley Children’s Hospital NICU.

### Inclusion criteria

Neonates with a birth weight of ≤ 1500 g or a gestational age of ≤ 30 weeks.

### Exclusion criteria

Presence of other congenital ophthalmic disease or cardiovascular disease; prior treatment with laser or intravitreal anti-VEGF agents.

A total of 50 low-birthweight, preterm neonates (100 eyes) were examined. 30 had follow-up exams approximately 2 weeks after their initial exam.

ROP Stage, Zone, and presence of plus disease in each eye were determined by an experienced retinal specialist (JDH) by indirect ophthalmoscopic examination as defined by the International Committee for the Classification of Retinopathy of Prematurity while masked to the PWU results^41^.

We performed PWU using a Vantage-128 (Verasonics, Inc., Kirkland, WA) research ultrasound system with a Verasonics L22-14vXLF linear array probe. The probe had an 18-MHz center frequency, 128 piezoelectric elements, a 12.8 mm aperture, and an elevation focal length of approximately 18 mm.

Scanning was performed at cribside in the NICU with the assistance of neonatal ophthalmic nurses (LP, OC). Exams were performed after the afternoon feeding, which made exams easier to perform and obviated concern regarding potential diurnal variation. Exams were performed by a single person (RHS) experienced in the performance of clinical ophthalmic ultrasonography. During the exam, the probe was placed in a viral barrier sheath (Sheathes3D; Sheathing Technologies, Inc., Morgan Hill, CA) filled with a cm of water, and coupled to the upper eyelid with GenTeal (Alcon Laboratories, Inc., Fort Worth, TX) gel with minimal pressure. Scans were performed in a horizontal plane encompassing the optic nerve. B-mode color-flow images were displayed in real time. Once the optic nerve and posterior vasculature were satisfactorily displayed, the system was triggered to acquire PWU compound data from six angled transmits over ± 9 degrees. Data were acquired continuously for 1.5 s at 3000 compound scans per second, and the stack of 4500 phase-resolved images stored for post-processing. We acquired three scans of each eye, repeating scans as necessary if degraded by eye or head movement. Examination of both eyes would typically take about 15 min, with much of this taken up by the 45 s wait to store each ~ 9 GB scan.

Acoustic intensity was compliant with FDA guidelines for ophthalmic diagnostic ultrasound standards^36^.

Doppler analysis was performed as follows: A singular value decomposition filter and a 10 Hz high-pass filter was applied to the data to suppress stationary or slowly moving structures, leaving only blood flow. This information was then used to produce a color flow image from which the CRA, CRV and short posterior ciliary arteries (SPCAs) were identified. We then sampled each vessel to produce spectrograms depicting flow pulsatile flow velocity over the 1.5 s of each scan. From this, after correcting for vessel angle with respect to the acoustic axis, we measured peak systolic velocity (PSV), end diastolic velocity (EDV) and mean velocity (MV), and computed the pulsatile index, PI = (PSV − EDV)/MV and resistive index, RI = (PSV − EDV)/PSV.

Statistical analysis was performed using IBM SPSS Version 29 (IBM Corp., Armonk, NY). Variation of Doppler values within eyes by exam was determined. ANOVA, with post-hoc Dunnett’s test, was used to compare Doppler parameters between ROP Stages for each vessel. We examined change of flow velocity with change in Stage between initial and followup exams. Because each eye was classified and imaged separately, we treated each eye as a separate case. However, recognizing that eyes of a given subject tend to be correlated, we additionally performed the analysis separately for right and left eyes.

ANOVA was also performed to evaluate the presence of preplus disease and Zone on flow.

Stepwise linear discriminant analysis was used to produce multivariate classification models to distinguish Stage 3 with respect to Stages 0–2. A priori probabilities for group membership were set equal. Classification accuracy was assessed and validated by a leave-one-out procedure. Receiver operator characteristic (ROC) analysis was performed to evaluate the effectiveness of classification functions.

We first produced a stepwise clinical model by entering sex, blood pressure (systolic, diastolic, mean, pulse pressure), GA and BW into the analysis (Model 1). Next, we produced a model using stepwise variable selection of all Doppler parameters (Model 2). Lastly, we produced a stepwise model simultaneously considering both clinical and Doppler parameters (Model 3).

## Data Availability

The datasets generated and analyzed during the current study are deposited in the Dryad repository: Plane wave Doppler determination of blood flow in retinopathy of prematurity [Dataset]. Dryad. 10.5061/dryad.612jm649r.
